# Enhancement of growth, antioxidative status, nonspecific immunity, and disease resistance in gibel carp (*Carassius auratus*) in response to dietary Flos populi extract

**DOI:** 10.1007/s10695-021-00992-z

**Published:** 2022-01-01

**Authors:** Xuhui Zhang, Zhiyuan Sun, Yuheng Wang, Yindi Cao, Guibin Wang, Fuliang Cao

**Affiliations:** 1 Co-Innovation Center for Sustainable Forestry in Southern China, Nanjing Forestry University, 159 Longpan Road, Nanjing Jiangsu, 210037 People’s Republic of China; 2 Department of Animal Husbandry and Veterinary Science, Jiangsu Polytechnic College of Agriculture and Forestry, Jurong Jiangsu, 212400 People’s Republic of China; 3 College of Resources and Environment, Qingdao Agricultural University, Qingdao, 266109 People’s Republic of China

**Keywords:** Gibel carp, *Flos populi* extract, Growth, Immune responses, Antioxidant, Pro-inflammatory cytokine

## Abstract

This study investigated the effects of dietary *Flos populi* extract (FPE) on the growth, antioxidation capability, innate immune response, and disease resistance in gibel carp. A total of 480 fish were fed with five different diets containing 0, 0.5, 1.0, 1.5, or 2.0 g kg^−1^ FPE (designated as control, D0.5, D1.0, D1.5, or D2.0 groups) for 45 days. The fish were challenged with *A. hydrophila* after the feeding trial. Compared with the control, the feed efficiency (FE), weight gain (WG), final body weight (FBW), and specific growth rate (SGR) were significantly improved in groups D1.0 and D1.5. Dietary FPE significantly increased serum superoxide dismutase (SOD), glutathione peroxidase (GPx), and catalase (CAT) activities, as well as glutathione (GSH) content. The contents of protein carbonyl (PCC) and malondialdehyde (MDA) in serum decreased significantly. Additionally, FPE supplementation in diets resulted in significant improvement in serum lysozyme (LZM) and myeloperoxidase (MPO) activities, as well as immunoglobulin M (IgM) and complement 3 (C3) concentrations. The hepatic antioxidant enzymes (CAT and SOD) activities increased, whereas content of MDA decreased in fish treated with dietary FPE than those of control both pre- and post-challenged. After 12 h-challenge, an obvious downregulation of hepatic Kelch-like-ECH-associated protein 1 (*Keap1*), splenic tumor necrosis factor-α (*TNF*-*α*), interleukin (*IL*)-*8*, *IL*-*1β*, and toll-like receptor 2 (*TLR2*) mRNA levels was observed in fish treated with dietary FPE, whereas hepatic *Nrf2* transcription level was upregulated compared to the control. Furthermore, compared to group D0.5, higher relative percent survival (RPS) was observed in gibel carp fed dietary 1.0–2.0 g/kg FPE. Our results reveal that FPE supplemented diet has a stimulatory effect on antioxidant capacity and nonspecific immune response, along with improved growth performance and enhanced resistance against *A. hydrophila* infection in juvenile gibel carp.

## Introduction

Gibel carp (*Carassius auratus gibelio*) is considered one of the most important cultivated freshwater fish species in China. It is easy to culture and breeds rapidly (Zhao et al. 2011). Owing to its rapid growth and high market demand, the annual yield of gibel carp was more than three million tons in 2018 (Fishery Bureau 2018). However, the rapid expansion of its production has brought about many serious problems, e.g., the development of the high-density culture and lack of the efficiency disease prevention strategy. As a result, such intensification may cause stressful conditions, which suppress the immune system, along with increase the susceptibility of fish to infectious diseases (Lueke et al. 2019; Harikrishnan et al. 2011; Cabello 2004). Furthermore, fish are often challenged by various stressors, which are originated from environmental changes and human activities (Conte 2004). These stressors result in poor antioxidation capability and lower immunity of gibel carp, leading to breeding failure and even catastrophic economic loss (Abdel-Tawwab et al. 2020). In animals, the use of antibiotics is no longer recommended due to their residues in fish, the increasing of high resistant of pathogenic bacteria, and negative impacts on the ecosystem that cause hazards to human health (Magouz et al. 2021). Consequently, the improvement of disease resistance and enhancement of fish antioxidant capacity and immunity has become an urgent need in healthy aquaculture (Hoseini et al. 2020; Magouz et al. 2021). Nowadays, plant-derived supplements are used as exogenous antioxidants and immunoprophylactics in aquaculture (Tan et al. 2018; Harikrishnan et al. 2011; Hai 2015).

In order to improve the fish health and disease management of aquatic animals, at least 60 herbal plants have been studied and implied in aquatic animals (Bulfon et al. 2015). Of these, natural plants have characteristics of growth-promoting, improving immune response, anti-inflammation, antibiotic capabilities, hepatoprotection, appetite stimulation, and anti-stress in fish (Citarasu 2010; Reverter et al. 2014). Most plants and their extracts contain phenolic glycosides, polyphenols, flavonoids, alkaloids, quinones, terpenoids, polysaccharides, and tannins or polypeptide compounds, which are effective alternatives to synthetic compounds and antibiotics (Hai 2015; Tan et al. 2018). Also, Chinese herbal medicine resources are excellent sources of nutrients for animals, which are low-cost, locally available, biodegradable, and environment-friendly, and can resist against a wide bacterial spectrum (Chang 2000; Reverter et al. 2014).

As an essential traditional Chinese medicine, *Flos populi* comes from the male inflorescence of *Populus canadensis* Moench or *Populus tomentosa* Carrière (*Salicaceae* family) (Committee 2010; Xu et al. 2014). It is traditionally used for fever reduction and detoxication. *Flos populi* has mainly been employed to treat various inflammatory and diarrhoeal diseases in East Asian countries for many years (Si et al. 2011; Xu et al. 2013, 2014; Zhao et al. 2014a, b; Hou et al. 2019). Chemically, *Flos populi* extract (FPE) is enriched with a blend of flavonoids and their glucopyranosides (quercetin, kaempferol, luteolin, apigenin, pinocembrin, chrysin, etc.), phenolics and cardiac glycosides (Si et al. 2010; Hou et al. 2019; Zhao et al. 2014a, b; Zhang et al. 2019). Besides, it contains polysaccharides, alkaloids, and organic acids. According to previous studies, FPE has antioxidant (Ni et al. 2019) and anti-inflammatory (Hou et al. 2019) activities both in vitro and in vivo. These researches confirmed the potential of FPE as an effective natural antioxidant or immunostimulant, but little information is available on the possible effects of dietary FPE supplementation on aquatic animals.

Gibel carp is a major fish species for freshwater aquaculture in China, in view of the importance of introducing new immune stimulants for the so-called green/antibiotic-free aquaculture. Accordingly, the current research was designed to explore the influence of diets containing FPE on growth, feed utilization, nonspecific immunity, antioxidant capability, and disease resistance in juvenile gibel carp.

## Materials and methods

### Experimental design and diet preparation

The formulation for the basal diets is presented in Table 1. The FPE was procured by Shaanxi Hengling natural biological products Co., Ltd (Xi’an, China) with 60% flavonoids and 10.1% phenolics, was included in the basal diet at levels of 0, 0.5, 1.0, 1.5, or 2.0 g/kg diet (Zhao et al. 2014a, b) at the expense of equal maize starch, respectively. The five groups were designated as control, D0.5, D1.0, D1.5, and D2.0, respectively. All ingredients used were ground into a powder that could pass through a 60-mesh sieve. After adding all the ingredients and stirring the mixture, all the diets were blended separately in a blender and then homogenized. Doughs with a diameter of 2.5 mm were wet-extruded by a granulator (SLP-45, Fishery Mechanical Facility Research Institute, Shanghai, China). After air drying (below 100 g/kg moisture of diet), all the diets were sealed individually and stored at − 20 °C for analysis.

**Table 1:** Chemical compositions and formulation of the basal diets (% dry matter)

Raw materials (%)	Percentage	Proximate analysis (% dry weight)	
Soybean meal	20.40	Dry matter	89.76
Rapeseed meal	26.00	Moisture	8.69
Cottonseed meal	18.00	Crude protein	35.064
Fish meal	10.00	Crude lipid	5.00
Maize starch	18.90	Ash	6.75
Salt	0.30		
Soybean oil	3.60		
Ca(H_2_PO_4_)_2_	1.80		
Vitamin-mineral mix^a^	1.00		
Total	100.00		

^a^Composition of vitamin-mineral mix (PREMIX PLUS) (quantity kg^−1^): vitamin: vitamin A (110 mg); vitamin D_3_ (20 mg); vitamin E (50 mg); vitamin K_3_ (20 mg); vitamin B_1_ (20 mg); vitamin B_2_ (20 mg); vitamin B_3_ (100 mg); vitamin B_6_ (20 mg); vitamin B_12_ (0.02 mg); folic acid (5 mg); d-calcium pantothenate (50 mg); inositol (100 mg); biotin (0.16 mg); vitamin C (140 mg); choline chloride (10 g); mineral: MgSO_4_·7H_2_O (4575 mg); FeSO_4_·7H_2_O (1250 mg); C_6_H_10_CaO_6_·5H_2_O (1750.0 mg); ZnSO_4_·7H2O (1110 mg); MnSO_4_·H_2_O (61.4 mg); CuSO_4_·5H_2_O (15.5 mg); CoSO_4_·7H_2_O (0.91 mg); KI (1.5 mg); Na_2_SeO_3_ (0.60 mg)

### Feeding trial conditions and fish

A batch of healthy juvenile gibel carp were obtained from a specialized aquatic fry farm (Nanjing, Jiangsu Province, China) and were reared in an indoor recirculating system. Before feeding experiments, 850 fish were all acclimated in fiber glass cylinders (200 L) under the experimental conditions for 2 weeks by feeding the control diet. During the acclimation, fish were fed up to apparent satiation thrice daily.

Two weeks later, fish had accustomed to the experimental conditions. After 24 h of fasting, the selected 600 healthy fish of uniform size (initial body weight (BW): 19.96 ± 0.06 g, initial protein content of fish: (12.60 ± 0.08)%) were distributed randomly into 20 tanks (200 L each) at a density of 30 fish per tank. Quadruplicate tanks were assigned to each dietary group in a random manner. Throughout the entire experimental period, fish were fed their respective diets per day at 07:30, 12:30, and 17:30. The trial lasted for 45 days. A lower pressure blower was used to supply sufficient oxygen.

During the trial, the water flowing rate (0.2 L/min), the water dissolved oxygen (7.38 ± 0.05 mg/l), pH (6.8–7.0), temperature (26.7 ± 1 °C), and ammonia (≤ 0.04 mg/l) of the tanks were recorded daily, along with a natural photoperiod. A portable analyzer (Aquacombo, China) was used to monitor the water physicochemical parameters inside the tanks daily. The feeding amount was adjusted according to BW measurement every two weeks. After 30 min of feeding, excess feed and fish feces were removed by siphon and about 33% of the tank water was renewed once a day to maintain water quality. The experimental program was approved by the Ethics and Animal Welfare of Nanjing Forestry University (Nanjing, China) (permit number: NJFU (Su) 2016–0024).

### Calculation of growth and feed utilization

Before sampling, all the fish were fasted for 24 h. The fish numbers in each tank were recorded, and the total weight of fish per tank was measured. Then, each fish was weighed to calculate SGR (specific growth rate, %/day), WGR (weight gain rate, %), FE (feed efficiency, %), FI (feed intake, g/day/individual fish), PRE (protein retention efficiency, %), and SR (survival rate, %) according to our previous publications (Zhang et al., 2020a).

### Challenge experiment

The obtention and culture of *A. hydrophila* were based on our previous study. The final bacterial concentration used for the challenge test is 2.4 × 10^7^ CFU/ ml according to the method described by Zhang et al. (2020a) and Ming et al. (2020).

Eight weeks post-feeding, after the fish were fasted for 24 h, then 23 healthy fish with similar body weight per tank were selected and were transferred into another labeled tank (200 L) under the same management conditions (23 fish per tank, 4 tanks per group) for challenge with bacterial septicemia pathogen *Aeromonas hydrophila.* Each fish was intraperitoneally injected with 200 μL of 2.4 × 10^7^ CFU/ml *A. hydrophila* suspension by medical syringe. After the injection, fish in each treatment were fed on the corresponding assigned diets during the whole challenge test. The fish in the original tanks were also injected intraperitoneally with 200 μL PBS as negative control. Twelve-hour post injection, 3 alive fish per tank were randomly selected for sampling. The fish were monitored for 96 h, and any dead fish were examined bacteriologically to confirm the presence of *A. hydrophila*. Numbers of fish alive (the sampled fish were excluded) were recorded 12–96 h post bacterial infection. The survival rate (%) was calculated as [(number of fish survived/ (initial number of fish − 3)] × 100. The relative percentage survival (RPS) (%) was calculated through the formula of Amend (Amend, 1981): RPS (%) = [1 − mortality (%) in treated group/mortality (%) in control group] × 100.

Sample collection.

When the feeding trial finished, after weighing the fish, 3 fish/tank were randomly selected and anesthetized on ice with diluted MS-222 (tricainemethanesulfonate, Sigma, WA, USA) at the concentration of 100 mg/L. Blood sample was then rapidly drawn from the caudal vein using 2 ml heparinized plastic syringes. The collected blood samples were centrifuged (3000 g, 4 °C, 15 min), and the plasma samples were obtained and stored at − 80 °C for subsequent analysis. After the fish were dissected, individual liver tissue was isolated and immediately put into liquid nitrogen. In the challenge test, 12-h post-injection, 12 alive fish per group were sampled for liver and splenic tissues. All the samples were stored at − 80 °C until further processing.

### Assay of biochemical and immune parameters in serum

The activities of serum alanine aminotransferase (ALT), aminotransferase (AST), alkaline phosphatase (AKP), lysozyme (LZM), and myeloperoxidase (MPO) were determined by the colorimetric method, and the contents of immunoglobulin M (IgM) and complement 3 (C3) were determined by the immunoturbidimetric method. The analyses were carried out using commercial kits (Nanjing Jiancheng Bioengineering Institute of China) with a Synergy2 multifunctional microplate reader (BioTek, USA).

### Measurements of hepatic and plasma antioxidant parameters

#### The preparation of hepatic homogenate

After sampling, hepatic sample was placed in a centrifuge tube; then, ice-cold phosphate-buffered saline (PBS: 0.064 mol/L, pH7.4) was added. The hepatic samples were homogenized by a hand-held homogenizer in an ice bath. 5 min later, the homogenate was then centrifuged at 5000 rpm at 4 °C for 10 min, then the supernatant was collected for the following analysis. The protein concentration in the supernatant was measured by the method of Bradford (1976).

#### Plasma and hepatic antioxidant capacity assay

The contents of glutathione (GSH), protein carbonyl content (PCC), and malonaldehyde (MDA), and the activities of catalase (CAT), glutathione peroxidase (GPx), and superoxide dismutase (SOD) were determined by the method of spectrophotometric, colorimetry, TBA, ammonium molybdate, DTNB, and hydroxylamine, respectively, using a commercial kit according to the manufacturer’s instructions (Nanjing Jiancheng Bioengineering Institute, Nanjing, China). Indicators of the hepatic antioxidant capacity were expressed as U/mg protein.

#### mRNA expression assay

Based on previous work (Zhang et al. 2020a), total RNA from the liver and spleen tissues of gibel carp were extracted using RNAiso Plus (TaKaRa, Dalian, China). Total RNA (1 μg) was reverse transcribed by a Thermo One-step RT-PCR kit in accordance with the manufacturer’s instructions. The relative expression levels of hepatic kelch-like erythroid cell-derived protein-1 (Keap1) and nuclear factor (erythroid-derived 2)-like 2 (Nrf2), and splenic tumor necrosis factor-α (TNF-α), interleukin -1β (IL-1β), IL-8, and toll-like receptor 2 (TLR2) of gibel carp were measured by real-time RT-PCR using TaKaRa RT-PCR Master Mix reagent and ABI OneStep Plus Sequence Detection System (Applied Biosystems, Foster City, CA, USA). Each sample was tested in duplicate. The primer sequences for Keap1, Nrf2, TNF-α, IL-1β, IL-8, and TLR2 were designed according to the published sequences and presented in Table 2. PCR amplification was performed under standard conditions. As the housekeeping gene, β-actin was used as an internal control gene to normalize the expression of target genes among treatments (Cao et al. 2018). After obtaining the threshold cycle (Ct) values of each sample, the relative mRNA expression levels of the above five genes were calculated by using the 2^−ΔΔ*CT*^ method. The mRNA expressions of the target genes were normalized to β-actin with the quantity of the control group scaled to 1.

**Table 2:** Accession numbers and sequences of primers used for real-time PCR

Genes	Sequences of primers (5′ ‐3′)	Accession No	Amplicon Size (pb)
*TLR2*	ACGTTTCTGCAAGCTACGGA	KC816575.1	171
	CGGGCTTCTGCTCCTTTTCT		
*IL*-*1β*	TTTGTGAAGATGCGCTGCTC	AB757758.1	133
	CCAATCTCGACCTTCCTGGTG		
*IL*-*8*	TGAAGGAATGAGTCTTAGA	KC184490.1	99
	AGCTCCACACTCTCTATGTG		
*TNF*-*α*	CGCTACTCTGATTCCTATGGC	KF500408.1	199
	GCTTTCGCTGTTGCCTTTCT		
*Keap1*	CTCACCCCCAACTTCCTGCAG	MG759382	150
	GATGAGCTGCGGCACCTTGGG		
*Nrf2*	CCCTTCACCAAAGACAAGCA	MG759384	128
	TTGAAGTCATCCACAGGCAG		
*β*-*actin*	TTGAGCAGGAGATGGGAACCG	JQ619774	115
	AGAGCCTCAGGGCAACGGAAA		

### Statistical analysis

All data were statistically analyzed using Statistical Package SPSS version 22.0 software and subjected to one-way ANOVA analysis. Duncan’s multiple range test was used to detect the significance of the difference in mean values among different treatments. Significant differences (*P* < 0.05) between the values obtained from pre and 12-h post-challenge tests were marked by asterisks above histogram bars using independent *t* test. The data were represented as mean ± standard error of mean (SEM). The significant difference level was set at *P* < 0.05.

## Results

### Evaluation of growth performance and nutrient efficiency

In comparison with the control, groups D1.0 and D1.5 presented the highest FBW, WGR, SGR, and FE of fish after 45 days of the feeding trial (Table 3) (*P* < 0.05). The D1.0 and D1.5 groups presented higher (*P* < 0.05) FBW, compared with the other two groups. The WGR in group D1.0 was higher (*P* < 0.05) compared with that of D0.5 and D2.0 groups, respectively. SR of the four FPE groups was significantly increased (*P* < 0.05), in comparison with the control. No significant difference (*P* > 0.05) of FI and PRE in gibel carp was found among the five groups.

**Table 3:** The effects of dietary *Flos populi* extract on feed utilization and growth performance of gibel carp

Items	Dietary treatments^1^				
	Control	D0.5	D1.0	D1.5	D2.0
IBW (g/fish)	19.98 ± 0.13	20.13 ± 0.16	20.04 ± 0.18	20.07 ± 0.17	19.98 ± 0.15
FBW (g/fish)	40.62 ± 0.37^b^	41.74 ± 0.53^b^	43.26 ± 0.44^a^	43.43 ± 0.42^a^	41.27 ± 0.39^b^
WGR (%)	103.30 ± 7.48^c^	108.27 ± 4.83^bc^	115.54 ± 5.03^a^	117.34 ± 4.91^ab^	106.53 ± 4.69^bc^
SGR (%/d)	1.27 ± 0.22^b^	1.31 ± 0.28^ab^	1.37 ± 0.19^a^	1.40 ± 0.28^a^	1.30 ± 0.17^ab^
FI (g/d/ ind.)	0.57 ± 0.01	0.56 ± 0.02	0.57 ± 0.01	0.55 ± 0.01	0.56 ± 0.02
FE (%)	64.66 ± 4.34^b^	68.90 ± 4.01^ab^	72.73 ± 3.05^a^	75.83 ± 5.20^a^	67.87 ± 5.32^ab^
PRE (%)	30.14 ± 0.41	31.59 ± 0.52	32.43 ± 0.45	31.98 ± 0.53	31.59 ± 0.66
SR (%)	96.00 ± 0.03^b^	100.00^a^	100.00^a^	100.00^a^	100.00^a^

Values were expressed as means ± SEM (*n* = 4) and in the same row no sharing the same letter denote significant differences (*P* < 0.05)

*IBW*, initial mean body weight; *FBW*, final mean body weight; *WGR*, weight gain rate; *SGR*, specific growth rate; *FI*, feed intake; *FE*, feed efficiency; *PRE*, protein retention efficiency; *SR*, survival rate

### Biochemistry assay in serum

ALT activities of fish in groups D1.0, D1.5, and D2.0 were considerably lower than that of the control group (Table 4). AST activities in the four FPE groups were all significantly lower (*P* < 0.05) than those of in the control group. In contrast, AKP activities of groups D1.0, D1.5, and D2.0 were higher (*P* < 0.05) by comparison with the control.

**Table 4:** Effects of dietary *Flos populi* extract on biochemical parameters in serum of gibel carp

Items	Dietary treatments^1^				
	Control	D0.5	D1.0	D1.5	D2.0
ALT (U/L)	22.05 ± 0.48^a^	20.40 ± 0.32^ab^	18.05 ± 0.14^b^	17.95 ± 0.21^b^	18.62 ± 0.19^b^
AST (U/L)	146.73 ± 3.54^a^	118.04 ± 5.60^b^	116.62 ± 3.30^b^	106.53 ± 6.73^b^	112.32 ± 2.55^b^
AKP (U/L)	104.88 ± 5.98^b^	110.13 ± 6.44^b^	128.20 ± 3.83^a^	126.79 ± 5.02^a^	124.97 ± 3.28^a^

Values were expressed as means ± SEM (*n* = 8) and in the same row no sharing the same letter denote significant differences (*P* < 0.05)

*ALB*, albumin; *AKP*, alkaline phosphatase; *AST*, aspartate aminotransferase; *ALT*, alanine aminotransferase; *HSI*, hepatosomatic index

### Serum antioxidant and immunological parameters

**Fig. 1:**
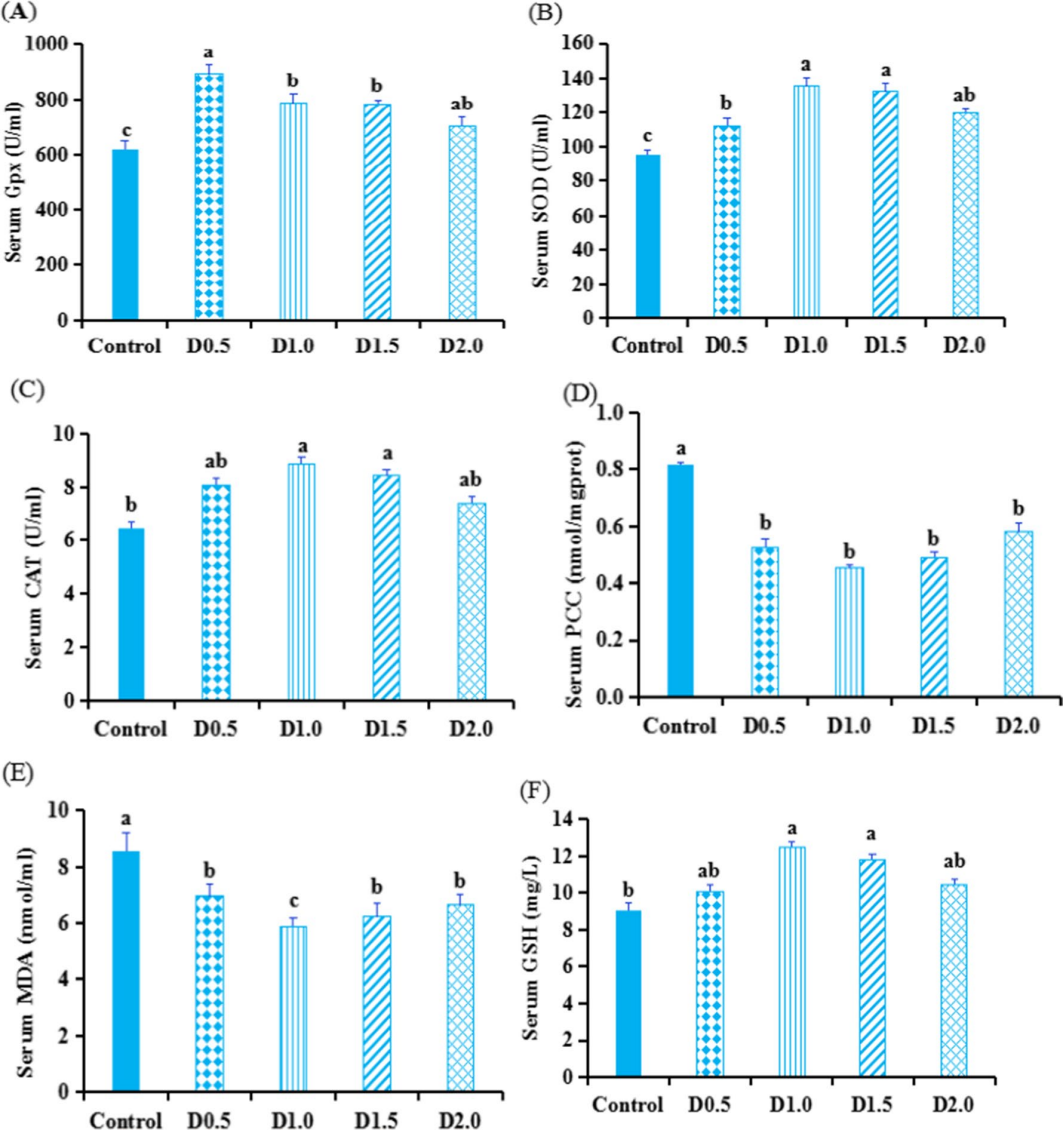
Influences of dietary *Flos populi* extract on serum GPx (A), SOD (B), and CAT (C) activities, and PCC (D), MDA (E), and GSH (F) contents in serum of *gibel carp*. Values are presented by mean ± SEM (*n* = 8). The bars marked with different lower letter denote significant difference between treatments (*P* < 0.05)

**Fig. 2:**
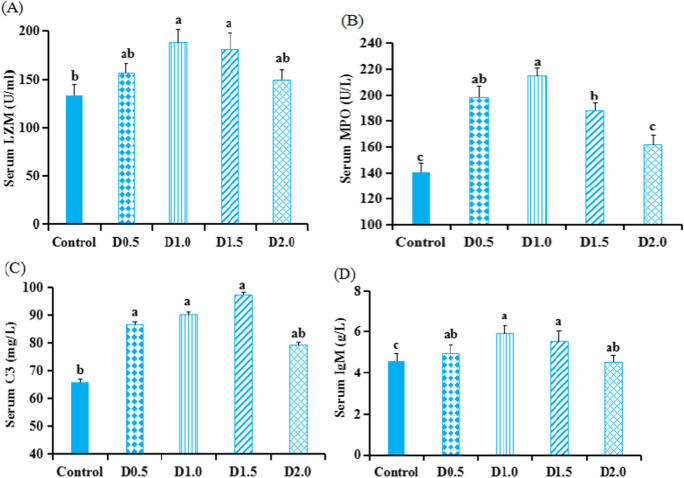
The activities of LZM (A) and MPO (B) activities and C3 (C) and IgM (D) contents in the serum of *gibel carp* fed dietary *Flos populi* extract for 45 days. Values are presented by mean ± SEM (*n* = 8). Bars not sharing a common superscript denote significant difference between treatments (*P* < 0.05)

Figure 1 and Fig. 2 show the serum antioxidant and immunological related parameters of gibel carp after 45 days of feeding trial.

### Serum antioxidant related parameters

Activities of GPx and SOD in serum increased in the treatment groups (Fig. 1). compared with the control group. At the same time, serum CAT activity of groups D1.0 and D1.5, as well as serum GSH content, showed a significant (*P* < 0.05) increase. In contrast, the serum MDA and PCC contents showed the opposite trend to SOD and GPx contents (*P* < 0.05 or *P* < 0.01).

### Serum immunological related parameters

As presented in Fig. 2, in comparison with the control, serum LZM activity was enhanced remarkably (*P* < 0.05) in D1.0 and D1.5 groups. Fish fed FPE supplemented diets displayed increased serum MPO activity (*P* < 0.05) and IgM concentrations (*P* < 0.01). In comparison with the control group, the serum C3 levels were higher (*P* < 0.05) in D0.5, D1.0, and D1.5 groups.

### Hepatic antioxidant capability

**Fig. 3:**
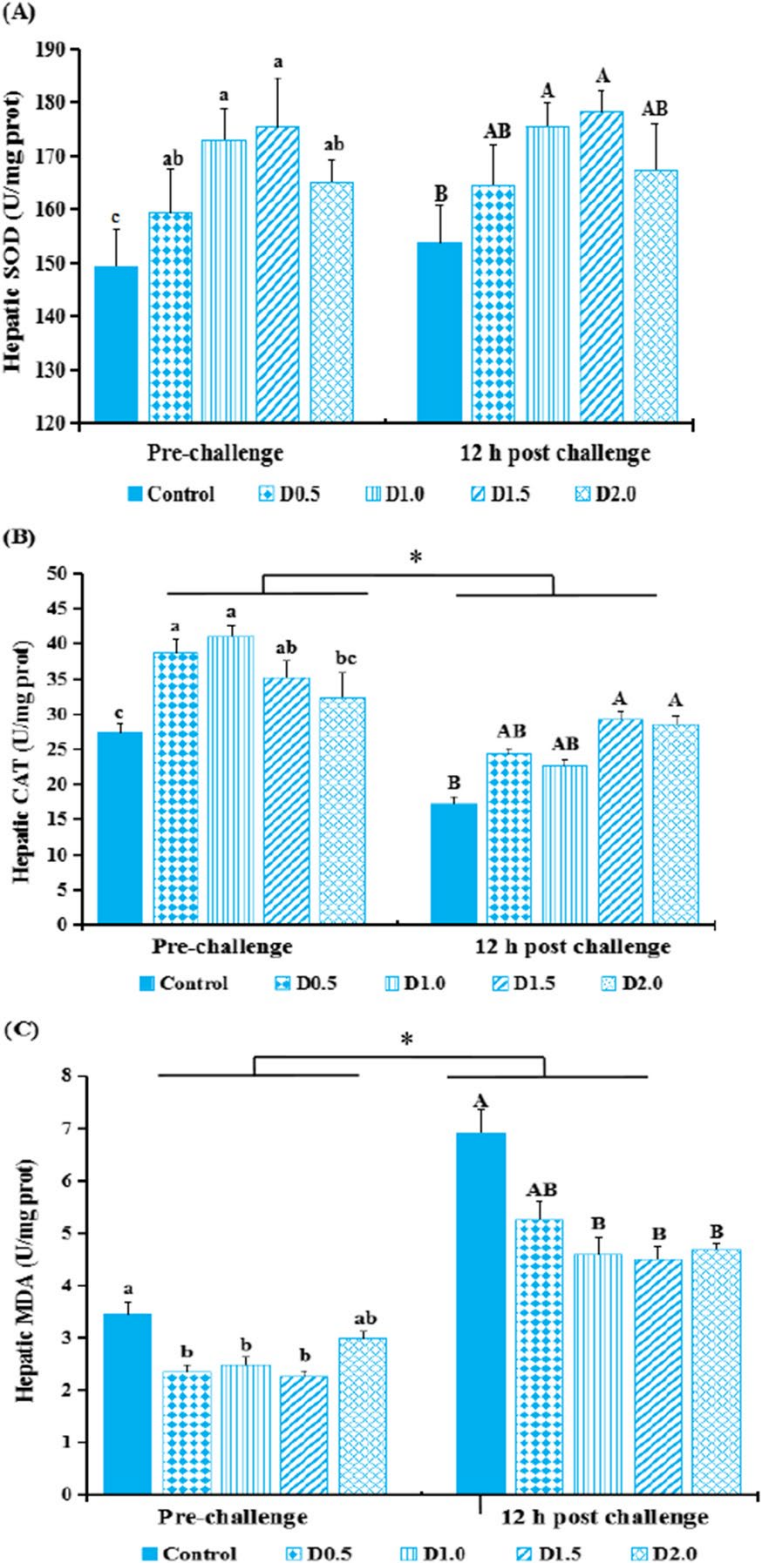
The activities of SOD (A), CAT (B) and contents of MDA (C) in the liver of gibel carp fed dieatry *Flos populi* extract pre-challenge or 12-h post-challenge*.* The values are presented as mean ± SEM (*n* = 8). Different lowercases above the bars denote significant differences between treatments in the pre-challenge test (*P* < 0.05). Different capital letters above the bars denote significant differences between treatments in 12-h post-challenge (*P* < 0.05). * means that there are significant differences between pre-bacterial challenge and 12-h post- bacterial challenge (*P* < 0.05)

Figure 3 presents the hepatic activities of SOD and CAT, as well as content of MDA of fish before or 12-h post-challenge tests. Before the challenge test, hepatic SOD activity in the FPE treated fish showing a remarkable enhancement compared with the control (*P* < 0.01). CAT activities of groups D0.5, D1.0, and D1.5 were significantly higher (*P* < 0.05) compared with the control. MDA content is the opposite of CAT activity.

At 12-h post-bacterial challenge, hepatic CAT activity decreased while the content of MDA increased (*P* < 0.05) relative to pre-infection levels. The hepatic MDA content in D1.0, D1.5, and D2.0 groups showed a significant decrease (*P* < 0.05), compared with the control. There was a remarkable increment (*P* < 0.05) in the activity of SOD in the groups D1.0, D1.5, and D2.0, as well as in the activity of CAT in the groups D1.5 and D2.0.

### Genes expression in liver and spleen

**Fig. 4:**
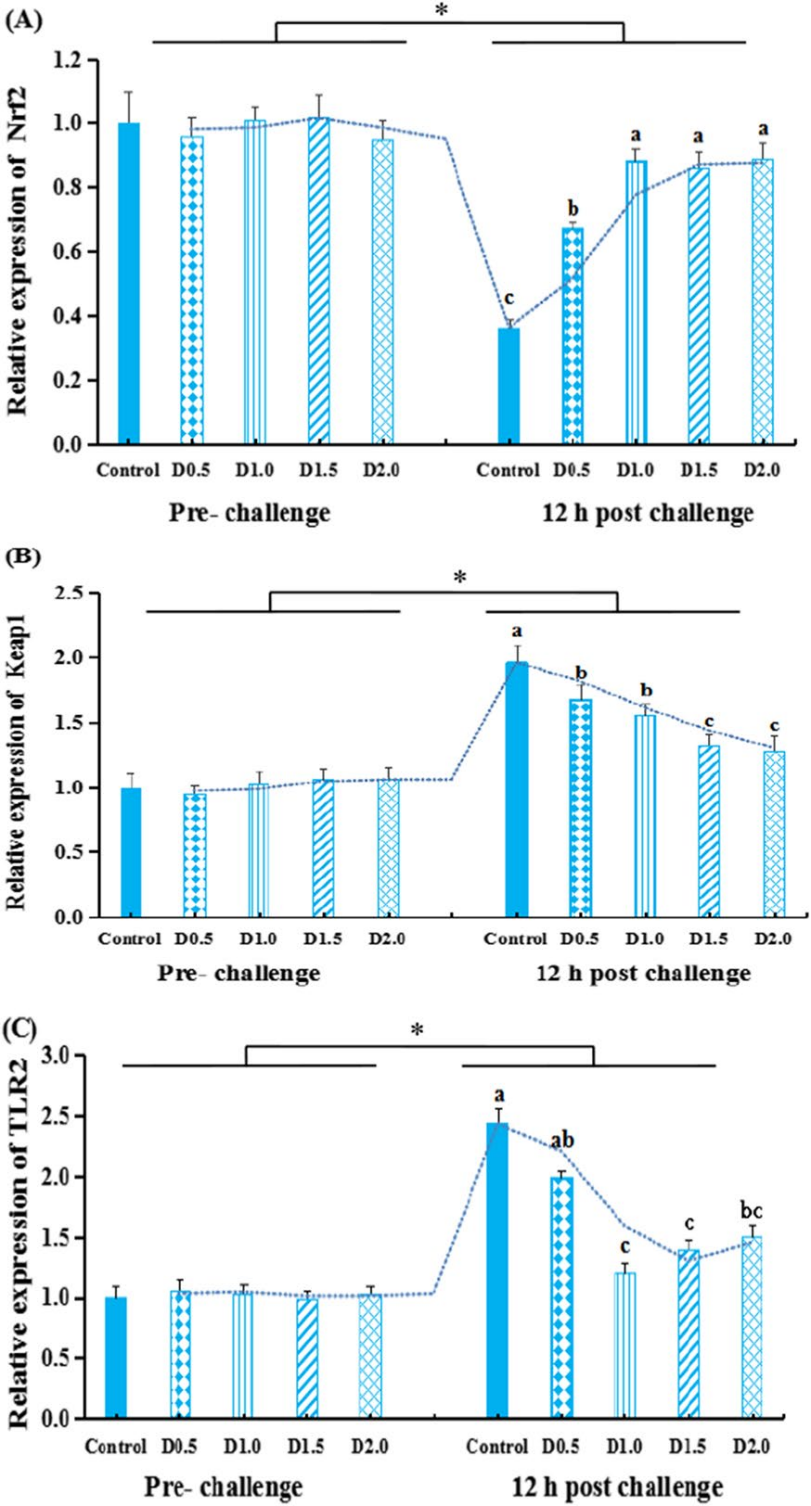

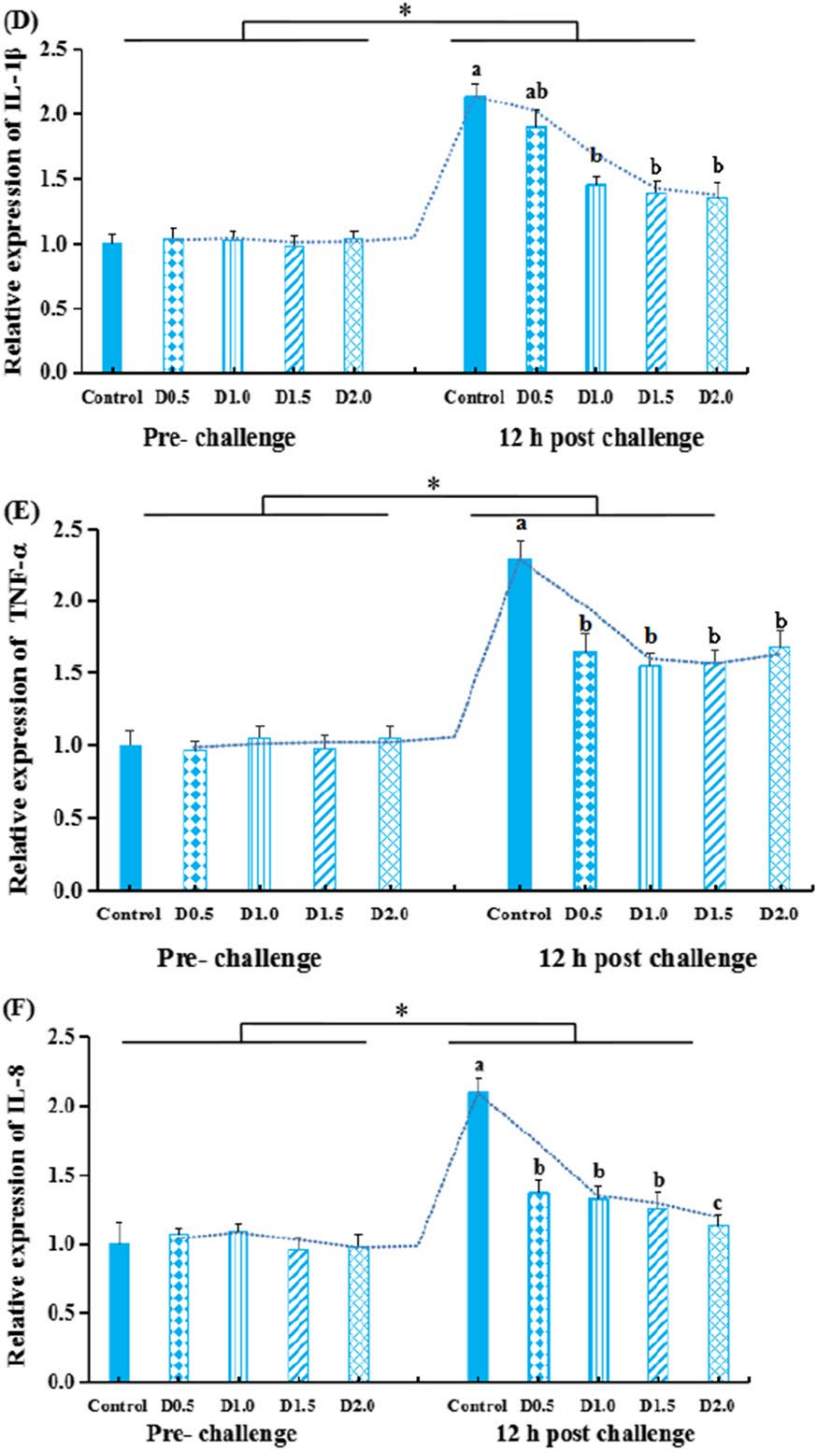
The transcriptional levels of *Nrf2* (A), *Keap1* (B) in the liver, and *TLR2* (C), *IL-1β* (D), *TNF*-*α* (E), *IL*-*8(F)* in the spleen of gibel carp fed dietary *Flos populi* extract pre- the challenge or 12-h post-challenge*.* The values are shown as mean ± SEM (*n* = 8). * means that there are significant differences between pre-bacterial injection and 12-h post-challenge (*P* < 0.05). The bars with different lowercases are significantly different (*P* < 0.05)

Figure 4(A–F) presents the transcriptional levels of antioxidant-related genes (*Nrf2* and *Keap1*) in liver and immune-related genes (*TLR2*, *TNF-α*, *IL*-*8*, and *IL*-*1β*) in the spleen of gibel carp before challenge and 12-h post-challenge. Before the challenge, no difference (*P* > 0.05) was observed on the expression of *Nrf2*, *Keap1*, *TLR2*, *TNF*-*α*, *IL*-*8*, and *IL*-*1β*.

At 12-h post-challenge, gene expressions of hepatic *Keap1* and splenic *TLR2*, *IL*-*1β*, *TNF*-*α*, or *IL*-*8* were remarkably upregulated in the injected fish than that of the pre-challenged one (*P* < 0.05). The hepatic *Nrf2* expression was inhibited by the 12-h post-challenge relative to pre-infection levels (*P* < 0.05). However, post the bacterial challenge, fish in FPE groups had lower expressional levels of Keap1, TNF-α, and IL-1β than those of fish in the control group (*P* < 0.05). The opposite was true for the expression of hepatic *Nrf2* (*P* < 0.01). Furthermore, groups D1.0, D1.5, and D2.0 also exerted a preventive effect on the increased levels of *TLR2* and *IL*-*8* in the spleen of post-challenged fish (*P* < 0.01).

### Cumulative survival rate of challenged fish

**Fig. 5:**
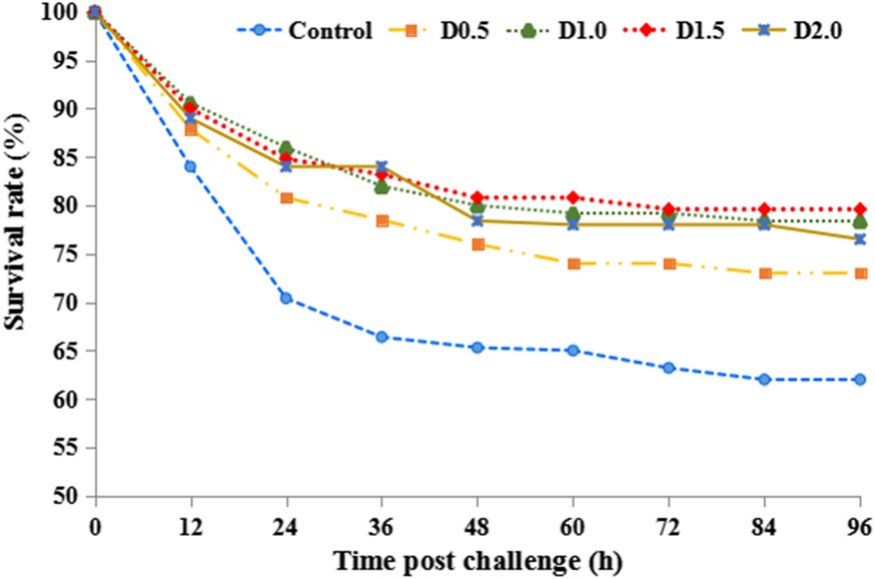
Effect of dietary *Flos populi* extract on the cumulative survival rate of gibel carp 96 h after injection with *A. hydrophila*

As shown in Fig. 5, after 96 h observation, no mortality was observed in negative group after injection with PBS. The results revealed that dietary FPE provision could enhance the resistance of gibel carp against *A. hydrophila* infection. The survival rates of the fish post-challenge were 62.00% (control group), 73.00% (D0.5 group), 78.40% (D1.0 group), 79.60% (D1.5 group), and 76.50% (D2.0 group), respectively. The RPS of the four treatment groups vs. the control group was 28.95%, 43.16%, 46.32%, and 38.16%, respectively. Furthermore, groups D1.0, D1.5, and D2.0 showed higher RPS (*P* < 0.05) compared with group D0.5. Typical symptoms of hemorrhagic septicemia were observed in dying or dead fish. *Aeromonas hydrophila* colonies were also isolated from dead fish.

## Discussion

### Growth and feed utilization

The proposed hypothesis of the present study is that dietary FPE is expected to improve the growth and health of gibel carp. Medicinal herb feed additives can improve feed conversion and digestibility by stimulating appetite, increasing digestive enzyme activity (Adel et al. 2015; Hai 2015). The results of this study suggested that the FBW, WGR, SGR, and FE were improved by dietary FEP at 1.0–1.5 g/kg diet. The growth-promoting effect in this study is probably attributed to the protective role of FEP, which defends the intestinal epithelium layer leading to high secretion of mucus that facilitates the cross of digested nutrients through villi until reaching the bloodstream (Zhu, 2020). A previous study showed that FPE possessed the properties of anti-diarrheal and antibiotic in vivo or in vitro (Xu et al. 2013). Maybe this is the reason for further explaining the improved feed utilization and decreased mortality. Adel et al. (2021) illustrated that polyphenols and natural antioxidants in medicinal herbs are involved in the stimulation of digestive enzymes in fish. Besides, the polyphenols in FPE have an antibacterial effect, which can limit and control the colonization and growth of pathogenic bacteria in fish intestines and allow the beneficial bacteria to digest the nutrients by the secreted digestive enzymes (Hai 2015; Wang et al. 2020; Mehrabi et al. 2020).

### Serum biochemistry

As the most important aminotransferases, ALT and AST are non-functional enzymes, which mainly exist in fish liver and kidney (Ghelichpour et al. 2020). Increased levels of AST and ALT are a sign of digestive function and liver damage in fish (Mirghaed et al., 2019; Ghelichpour et al. 2020). AKP is an alkaline phosphatase enzyme with antibacterial properties (Iger and Abraham 1990) and a valuable indicator of macrophage activation (Gobi et al. 2016). Therefore, an increased activity of AKP suggests the improvement of immune status (Hoseinifar et al. 2018; Roosta et al. 2014). In serum, the AKP activity increased while ALT and AST levels decreased by the supplementation of 1.0–2.0 g/kg FPE, which may be attributed to the protective capabilities of FPE. FPE may enhance the stability of cell membrane stabilization and protect tissues from free radical-induced toxic damages, which result in decreased levels of AST and ALT, as well as increased AKP activity. Similar results were reported in studies on Golden pompano (*Trachinotus carolinus*) fed with dandelion (*Taraxacum* spp.) extract (Tan et al. 2017).

### Antioxidant capability

Excessive ROS can induce biomolecular damage, including lipid, DNA, and protein, leading to lipid peroxidation along with protein carbonylation (Zheng et al. 2019). Fish antioxidant defense system mainly consists of antioxidants of low molecular weight and antioxidant enzymes to counter ROS (Martınez-Alvarez et al. 2005). Superoxide radicals are decomposed into harmful hydrogen peroxide by SOD; then, the product is decomposed into oxygen and water by GPx and CAT (Mirghaed et al. 2020). Glutathione can protect cells from oxidative damages, which is regarded as the main endogenous antioxidant scavenger (Liang et al. 2011). The contents of PC and MDA are always used to assess protein oxidation and lipid peroxidation during the oxidative stress (Jiang et al. 2016). Generally, lots of antioxidant potential of natural products were attributed to their rich flavonoid and polyphenolic compounds. FPE has been shown to possess both scavenging free radicals and stimulating antioxidant enzymes in vivo and in vitro. In the present study, both antioxidant-related enzymatic activities (CAT, SOD, and GPx) and antioxidant-related metabolites (e.g., GSH) from unchallenged fish were higher in the FPE diet groups. In the same time, reduced contents of MDA and PCC in serum were observed. The trend of hepatic activities of SOD and CAT, as well as contents of MDA, was consistent with those in serum in the pre-challenge test.

*hydrophila* challenge can induce ROS (Meydani et al. 1995), which can cause oxidative stress in fish. A variety of herbaceous plants have been shown to have a strong ability to scavenge free radicals, because of the multiple phenolic hydroxyl groups in their structure (hydrogen donors and singlet oxygen quenchers) and indirectly raise the capability to resist stress (Hoseinifar et al. 2017; Alagawany et al. 2020; Tian et al. 2019). Both phenolics and flavonoids are effective scavengers of most oxidizing molecules and other free radicals implicated in several diseases (Mbokane and Moyo 2018). FPE contains the blend of flavonoids and their glucopyranosides, and phenolics can resist oxidation, remove the free radical, and improve the immune function (Tian et al. 2019). Our study indicated that the hepatic SOD activity decreased, while MDA content tended to increase at 12-h post-challenge, but no matter before or after challenge, the treatment groups increased the hepatic CAT and SOD activities and mitigated the increment of MDA production compared with the control, which enhanced the antioxidant capacity.

In fish, Nrf2 signaling is the main pathway to regulate the antioxidant capacity. Meanwhile, as an Nrf2-binding protein, Keap1 can depress the translocation of Nrf2 to the nucleus (Li et al. 2008; Xu et al. 2018; Wang et al. 2020). Antioxidant proteins, including GPx, SOD, and CAT, were regulated by Nrf2 (Niu et al. 2019; Kobayashi and Yamamoto 2005). Under oxidative stress, Nrf2 is released from Keap1, translocates to the nucleus, and induces overexpression of antioxidant genes to restore redox homeostasis (Kaspar et al. 2009). In this study, the changes of antioxidant proteins levels were consistent with that of the Nrf2 gene, suggesting that Nrf2 is required for FPE during the induction of antioxidant capacity. Furthermore, the decreased expression of Nrf2 and increased expression of Keap1 in the FPE treated fish liver were alleviated, which indicates that the antioxidant mechanism of FPE may be through the Nrf2 signaling pathway by up-regulating Nrf2 expression and downregulating Keap1 expression. The results are also consistent with previous reports that doses of *Yucca schidigera* extract (Wang et al. 2020) and lotus leaf (Zhu et al. 2019) can increase antioxidant capacity by downregulating Keap1 mRNA expression, and further promoting Nrf2 translocation to the nucleus in fish.

### Immune response

Phenolic and flavonoid-rich plant extracts can be used in feed to improve the immunity status of fish (Jia 2019; Tan et al. 2020). According to our previous study, the antioxidant defense system of fish is closely related to immune system and health status (Zhang et al. 2020b). Myeloperoxidase catalyzes the breakdown of hydrogen peroxide (one of oxidative radicals) into hypochlorous acid, which possesses the antimicrobial activity playing an essential role in the defense of an organism (Dalmo et al. 1997). Furthermore, the humoral components, such as immunoglobulins, LZY, and complement, play a vital role in innate (or) nonspecific and specific immunity in fish. Lysozyme can inhibit the incursion of detrimental bacteria by decomposing their cell wall (Alexander and Ingram 1992). Complement plays a vital role in antibody production, microbial killing, inflammatory reaction, phagocytosis, and immune complex clearance (Holland and Lambris 2002). Complement 3 is a central molecule in the complement system, which can regulate the immune responses of B and T cells (Yano 1995).

Immunoglobulin M (IgM) is one of the three major isotypes of immunoglobulin, which responds to pathogens both in local and systemic pathogens (Salinas et al. 2011). Our data revealed that dietary FPE increased the levels of LZM and MPO (antimicrobial enzymes) in gibel carp and then enhanced the innate immunity and resistance to invading pathogens. The increased production of plasma LZM in the FPE treated fish might be due to the increased neutrophil count in blood (Hoseinifar et al. 2019). Also, the increased C3 content may be due to the induction of EFP to its production in the liver (Ghelichpour et al. 2017). The higher IgM concentration in the four FPE groups also showed that IgM could be a targeted molecular mechanism for FPE to enhance the immune function of fish. The present results indicated that the FPE supplementation elicited a nonspecific immune response, which is consistent with published literature that plant extracts increased MPO (Divyagnaneswari et al. 2007; Christybapita et al. 2007; Kaleeswaran et al. 2011; Gobi et al. 2016) and LZM activities (Talpur and Ikhwanuddin 2013; Parayet al. 2020), as well as the complement and IgM concentrations (Wang et al. 2020; Zhu et al. 2019; Abdel-Tawwab et al. 2018; Tan et al. 2017; Paray et al. 2020).

### Anti-inflammation

Fish immunity is closely associated with inflammatory response and antioxidant status (Zhao et al. 2013). *Flos populi* has been used to treat inflammation in traditionally. We measured the levels of splenic TLR2 signaling pathway related genes and pro-inflammatory cytokines to corroborate the anti-inflammatory properties of FPE during *A*. *hydrophila* infection. Fish experimentally infected with *A. hydrophila* presented with higher splenic TLR2, TNF-α, IL-8, and IL-1β expression levels by the previous study for gibel carp infected with *A. hydrophila* (Cao et al. 2018). Inflammatory cytokines contribute to orchestrate the anti-infectious innate immune response during infectious processes, but overzealous production of inflammatory cytokines induces cytokine storm, which is deleterious and contributed to mortality (Cavaillon 2018). Four dietary FPE concentrations were able to prevent the increase in splenic TNF-α, IL-8, and IL-1β expression levels elicited by infection. This demonstrated the potential anti-inflammatory effects of FPE. Similarly, Hou et al. (2019) reported the release of IL-1β, IL-6, and TNF-α, which were associated with inflammation, was attenuated by the compound from extract of *Flos populi* in LPS-stimulated RAW 264.7 cells. This effect was associated with the presence of flavonoids, which contains γ-sitosterol, quercetin, apigenin, pinostrobin, kaempferol, luteolin, apigenin-7-O-d-glucoside, and kaempferol-3-O-β-glucoside (1–2)-[α-rhamnopyranoside(1–4)]-β-glucoside (Xu et al. 2014).

TLR signaling pathway in fish immune tissue is activated significantly after the invasion of *A. hydrophila* (Mu et al. 2010; Zhang et al. 2018; Lü et al. 2015). Under stress, TLRs-MyD88 signaling pathway activation can further induce NF-κB to produce inflammatory cytokines (Akira and Takeda 2004). In fish, TLR2 was confirmed to play a vital role in innate immune reactions (Fan et al. 2015; Zhang et al. 2017; Liu et al. 2016). Our previous study showed that *Moringa oleifera* Lam leaves rich in flavonoids and polyphenols can normalize the transcriptional levels of pro-inflammatory cytokines via regulating TLR2 signaling (Zhang et al. 2020a). *Radix Bupleuri* extract treatment reduced inflammatory response and IL-1β, TNF-α, and IL-8 mRNA levels by inhibiting TLRs-MyD88-NF-κB signaling pathway (Jia et al. 2019a, b). In line with previous reports, our results indicate that TLR2-MyD88-NF-κB signaling pathway plays the role of protection against oxidative damage and anti-inflammatory response of FPE in gibel carp. These results suggest that the feeding of FPE to gibel carp exerted anti-inflammatory and immunomodulatory properties after bacterial infection. This may be due to the presence of high content of flavonoids, alkaloids, organic acids, phenols, and amino acids, which can help in building the immunity capacity (Abdel-Razek et al. 2019).

Pathogen infection is usually accompanied by an increase in free radical production (Liu et al. 2012). As a vital transcription factor, Nrf2 not only is responsible for regulating the anti-oxidative capacity but also plays a critical role in attenuating pro-inflammatory stimulation (Kim et al. 2010). Nrf2-mediated antioxidant response is consistent with those of TLR2 mediated anti-inflammatory response, as well as the defensive components (MPO, LZM, IgM, and C3) in serum, as evident in this study by the increased RPS against *A. hydrophila* of gibel carp after 96-h challenge with *A. hydrophila.* Antibacterial activity of FPE was shown previously that against *Salmonella typhi*, *Shigella flexneri*, and *Escherichia coli* in vitro (Xu et al. 2013). However, there was no study about FPE on disease resistance of fish. This may be due to its role in enhancing the defense system with increasing the different immune parameters exhibiting its antibacterial activity (Abdel-Razek et al. 2019). Consistent with the current study, beneficial effects of other medicinal herbs on disease resistance were also shown in previous studies (Gobi et al. 2016; Tan et al. 2017; Abdel-Razek et al. 2019; Mehrabi et al. 2020; Zemheri-Navruz et al. 2019; Adel et al. 2021).

## Conclusions

Taken together, our results indicated that dietary FPE could notably improve antioxidant capability, feed utilization, nonspecific immune, and disease resistance of gibel carp against *A. hydrophila*, as well as mitigate the excessive inflammatory response of gibel carp. Therefore, FPE at 1.0 and 1.5 g kg^−1^ levels is recommended as a functional feed additive for gibel carp.

## Data Availability

The data that support the findings of this study are available from the corresponding author upon reasonable request.
